# Early Prosodic Acquisition in Bilingual Infants: The Case of the Perceptual Trochaic Bias

**DOI:** 10.3389/fpsyg.2016.00210

**Published:** 2016-02-23

**Authors:** Ranka Bijeljac-Babic, Barbara Höhle, Thierry Nazzi

**Affiliations:** ^1^Laboratoire Psychologie de la Perception, Université Paris Descartes – CNRSParis, France; ^2^Université de PoitiersPoitiers, France; ^3^Universität PotsdamPotsdam, Germany

**Keywords:** bilinguals, infants, language, prosody, lexical stress, dominance effects

## Abstract

Infants start learning the prosodic properties of their native language before 12 months, as shown by the emergence of a trochaic bias in English-learning infants between 6 and 9 months (Jusczyk et al., 1993), and in German-learning infants between 4 and 6 months (Höhle et al., 2009, 2014), while French-learning infants do not show a bias at 6 months (Höhle et al., 2009). This language-specific emergence of a trochaic bias is supported by the fact that English and German are languages with trochaic predominance in their lexicons, while French is a language with phrase-final lengthening but lacking lexical stress. We explored the emergence of a trochaic bias in bilingual French/German infants, to study whether the developmental trajectory would be similar to monolingual infants and whether amount of relative exposure to the two languages has an impact on the emergence of the bias. Accordingly, we replicated Höhle et al. (2009) with 24 bilingual 6-month-olds learning French and German simultaneously. All infants had been exposed to both languages for 30 to 70% of the time from birth. Using the Head Preference Procedure, infants were presented with two lists of stimuli, one made up of several occurrences of the pseudoword /GAba/ with word-initial stress (trochaic pattern), the second one made up of several occurrences of the pseudoword /gaBA/ with word-final stress (iambic pattern). The stimuli were recorded by a native German female speaker. Results revealed that these French/German bilingual 6-month-olds have a trochaic bias (as evidenced by a preference to listen to the trochaic pattern). Hence, their listening preference is comparable to that of monolingual German-learning 6-month-olds, but differs from that of monolingual French-learning 6-month-olds who did not show any preference (Höhle et al., 2009). Moreover, the size of the trochaic bias in the bilingual infants was not correlated with their amount of exposure to German. The present results thus establish that the development of a trochaic bias in simultaneous bilinguals is not delayed compared to monolingual German-learning infants (Höhle et al., 2009) and is rather independent of the amount of exposure to German relative to French.

## Introduction

The majority of children around the world grow up in bilingual families or countries, acquiring more than one language at a time (Grosjean, 2010). Despite being exposed to a more complex language situation, bilingual children succeed in the task of simultaneously learning their two native languages and pass the language development milestones at roughly the same ages as their monolingual peers (Byers-Heinlein et al., 2010). However, this does not mean that language acquisition proceeds in exactly the same way in mono- and bilingual children. When discriminating phonetic contrasts present in only one of their languages, bilinguals usually succeed as monolinguals (Burns et al., 2007; Sundara et al., 2008; Albareda-Castellot et al., 2011; Sundara and Scutellaro, 2011), although a U-shaped curve not observed in monolinguals has been found for bilingual infants in some studies (Bosch and Sebastián-Gallés, 2003; Sebastián-Gallés and Bosch, 2009). In some language-related tasks, bilingual infants have shown an advantage over monolinguals at both 10 months (Garcia-Sierra et al., 2011; Bijeljac-Babic et al., 2012) and even 7 months of age (Kovács and Mehler, 2009). These data suggest that hearing two different languages provides bilingual infants with greater experience in processing a more variable input and develop cognitive flexibility in both linguistic and non-linguistic tasks.

While previous studies focused on early development of segmental phonology in this population, very little is known regarding how prosodic properties are processed and acquired by very young bilingual infants. The present study therefore explored how simultaneous bilingual infants acquire fundamental prosodic properties of their two native languages when these languages differ in the realization of lexical stress. Prior to presenting this work, we review what we know about early prosodic acquisition in bilingual infants compared to monolingual infants. At present, the bulk of the available data relates to early language discrimination or recognition, and processing of stress patterns.

Monolingual infants have been found to recognize their native language at birth (Mehler et al., 1988; Moon et al., 1993). Moreover, both newborn and 2-month-old monolinguals can discriminate languages only if they differ by their rhythmic properties (Christophe and Morton, 1998; Nazzi et al., 1998; Byers-Heinlein et al., 2010), while 3.5- to 5-month-old monolinguals can discriminate their native language from rhythmically similar ones (Nazzi et al., 2000; Butler et al., 2011; Molnar et al., 2013). Similarly, bilingual newborns prefer to listen to both of their languages over a rhythmically different one, and can discriminate them if they have different rhythms (Byers-Heinlein et al., 2010). By 3–5 months of age, they can discriminate their two native languages if they are rhythmically similar (Bosch and Sebastián-Gallés, 1997; Molnar et al., 2013). While some fine-grained differences in shift latencies and overall listening times (Bosch and Sebastián-Gallés, 1997) suggest that bilinguals might attend to their native languages differently than monolinguals, bilinguals appear to have highly similar language discrimination and recognition abilities as monolinguals, probably based on the processing of prosodic properties at the utterance level.

Regarding the processing of more local prosodic properties, several studies have compared stress pattern discrimination in French- vs. Spanish- (Skoruppa et al., 2009; Abboub et al., 2015) or German-learning (Friederici et al., 2007; Höhle et al., 2009; Bijeljac-Babic et al., 2012) monolingual infants. While lexical stress is found in most languages of the world, including Spanish, German, English, and Dutch, French does not use stress contrasts at the lexical level. However, French has fixed phrasal-final stress which is mostly marked by a lengthening of the phrase-final syllable (Hayes, 1995; Di Cristo, 1999; Jun and Fougeron, 2000, 2002). Moreover, stress is not realized acoustically in the same way in all languages: for example, duration appears to have a more important role in prosodic phrasing in French than in German (Féry et al., 2011); and both F0 and intensity are reliably higher for a syllable that has phrasal stress in German, while they can be dissociated in French (Vaissière and Michaud, 2006; Nespor et al., 2008). The fact that French only has phrase-final stress appears to have an impact on stress pattern discrimination by French-speaking adults: compared to both Spanish and German adults, French speakers show a reduced sensitivity to stress (that has sometimes been called “stress deafness”) when processing either words presented in isolation (Dupoux et al., 1997, 2001) or continuous sequences made up of nonsense syllables or nonlinguistic sounds (Bhatara et al., 2013, 2015; Boll-Avetisyan et al., 2015). This reduced sensitivity in French adults (which does not prevent them to use phrase boundaries as cues for segmentation, Michelas and D’Imperio, 2015) is particularly marked when the stimuli presented are characterized by speaker or segmental variability, suggesting that crosslinguistic differences emerge in experimental contexts in which the stimuli need to be processed at a phonological level, rather than at an acoustic or phonetic level.

When are these crosslinguistic differences set into place? Are infants growing up with different linguistic backgrounds differentially sensitive to stress patterns depending on whether they can process that information at the phonetic level (when presented with stimuli lacking segmental variability), or whether they have to process it at the phonological level (when presented with stimuli containing segmental variability)? Accordingly, previous studies tested infants in two different conditions. In the no segmental variability condition, infants were presented with different tokens of a single item (e.g., /gaba/) recorded either with a stress-initial (trochaic) or stress-final (iambic) pattern, allowing for discrimination based on lower-level acoustic properties. In the more challenging (segmental variability) condition, infants were presented with lists of segmentally different items (e.g., /datu/, /sapi/, /kiba/, /nuki/..) recorded with either a trochaic or an iambic pattern, such that discrimination was only possible if infants could abstract and generalize the stress patterns over segmental variability.

For monolingual infants, and in the absence of segmental variability, discrimination was found in Italian newborns (Sansavini et al., 1997), English-learning 1- to 4-month-olds (Spring and Dale, 1977), German-learning 4-month-olds (electrophysiological data: Friederici et al., 2007; behavioral data: Herold et al., 2008), and Spanish-learning 6-month-olds (Skoruppa et al., 2013). Importantly, it was also found in French-learning infants from 4 to 10 months of age (Friederici et al., 2007; Höhle et al., 2009; Skoruppa et al., 2009, 2013). This establishes early stress discrimination abilities in the absence of segmental variability that appears to be independent of native language experience. However, French-learning infants’ sensitivity to lexical stress declines between 6 and 10 months of age. While at 6 months, they could discriminate stress patterns following a 1-min familiarization with one pattern, at 10 months they needed 2 rather than 1 min of familiarization (Höhle et al., 2009; Skoruppa et al., 2009; Bijeljac-Babic et al., 2012). Thus, at 10 months, French-learning infants require more time to identify stress patterns, a developmental path reflecting early language-specific reorganization probably leading to the “stress deafness” found in French adults (Dupoux et al., 1997, 2001; Bhatara et al., 2013, 2015; Boll-Avetisyan et al., 2015).

Still for monolingual infants, what do we know about stress pattern discrimination in the presence of segmental variability? Such ability seems limited in young infants. Indeed, newborns were found to discriminate stress patterns when presented with lists of words varying on consonants (Sansavini et al., 1997) but not when the words varied on both consonants and vowels (Sansavini et al., 1994). Early limitations were further attested by Spanish- and French-learning 6-month-olds’ difficulty at discriminating stress patterns when presented with lists of segmentally different words (Skoruppa et al., 2013). Discrimination of stress patterns across segmentally varying words were found later in development, at 9 months in Spanish-learning infants, and at 8 and 12 months in English-learning infants (Skoruppa et al., 2009, 2011). Importantly, such discrimination abilities appear to be modulated by the native language: French-learning 9 to 10-month-olds failed to discriminate, thereby showing that they cannot process stress patterns across multiple segmentally varied items, whereas they can do so in tasks using only one item (Skoruppa et al., 2009; Abboub et al., 2015).

As for bilingual infants, only two studies explored stress pattern discrimination in either the absence (Bijeljac-Babic et al., 2012) or the presence (Abboub et al., 2015) of segmental variation. Given the earlier studies on monolingual infants showing that sensitivity to lexical stress changes during development as a function of the prosodic characteristics of the native language, these studies explored prosodic acquisition in bilingual infants learning two languages with different lexical stress pattern systems. In both studies, 10-month-old bilingual infants learning French (a language lacking lexical stress contrasts) and a language that has variable lexical stress (from a set of about 15 different languages) were found to discriminate stress contrasts, and to perform better than French-learning monolinguals of the same age. These findings thus establish that the presence of a second language with variable lexical stress maintains sensitivity to lexical stress in these bilingual infants learning French. This stress pattern discrimination in bilinguals was found in the absence as well as in the presence of segmental variability in the stimuli. Moreover, in these two studies, none of the bilingual infants were learning the language in which the stimuli had been produced (German for Bijeljac-Babic et al., 2012; Spanish for Abboub et al., 2015), demonstrating that these discrimination effects are not just based on the recognition of the exact acoustic cues used in their second language to mark lexical stress, but that they possibly reflect the sensitivity to abstract stress patterns.

These two studies also explored the effect of language dominance, in order to determine whether infants who receive less French input and more of the languages with lexical stress have better stress discrimination abilities. In Bijeljac-Babic et al. (2012), stress pattern discrimination was significant in the subgroup of infants dominant in the languages with lexical stress (receiving 70–80% of their input in these languages) but was only marginal in the balanced bilinguals (receiving 40–60% of their input in both languages), suggesting an effect of language dominance. However, in Abboub et al. (2015), no effect of language dominance was found: first, discrimination performance did not differ for French-dominant (hearing French 60 to 70% of the time in their input) versus not French-dominant infants (hearing French only 30 to 50% of the time), and second, there was no correlation between performance and percentage of German heard. Taken together, the two studies reveal only a weak impact of language dominance on prosodic processing, at least for discrimination abilities, suggesting that already a limited amount of exposure to a language with lexical stress allows the maintenance of discrimination abilities. Would the same hold for the acquisition of a prosodic property (namely the predominant stress pattern of words in the native language) that requires not only to discriminate stress patterns but also to conduct distributional analyses on the relative frequency of each pattern within the input?

In monolinguals, early language-specific developmental changes have been found. This was revealed by the emergence of a preference for trochaic over iambic items lacking segmental variability in German-learning infants between 4 and 6 months of age, the trochaic pattern being predominant in German (and English), while such bias was not found in French-learning 6-month-olds (Höhle et al., 2009). Still in monolinguals, but using lists of segmentally varied words, a trochaic bias was found to emerge in English-learning infants between 6 and 9 months of age (Jusczyk et al., 1993). However, a different pattern was found for Spanish (Pons and Bosch, 2010), a language with a relatively balance of trochaic (60%) and iambic (40%) words, but in which stress assignment is related to syllabic structure (95% of CVC.CV words are trochaic; 93% of CV.CVC words are iambic). Presented with lists of segmentally varying words, Spanish-learning 9-month-olds showed no stress pattern preference for CV.CV words, a trochaic preference for CVC.CV words and an iambic preference for CV.CVC words. Taken together, these results show that after 6 months of age, monolingual infants learning a language with variable lexical stress have learned the predominant stress pattern of their native language (and its link to syllabic structure). These findings further suggest that recognizing this pattern becomes more efficient in the following months, allowing infants to abstract the stress pattern from segmentally varying strings. On the other hand, monolingual infants learning French appear not to develop a trochaic bias, as no such bias is present in their linguistic input.

The above acquisition pattern thus raises the question of whether and when bilingual infants learning French and a language with a predominant lexical stress pattern develop a preference for that predominant pattern. The present study explored this issue, extending Höhle et al. (2009) to French/German bilingual infants, in order to determine whether by 6 months of age, these infants have a trochaic bias when listening to German stimuli in the absence of segmental variability, as has been found for their German- but not their French-learning age mates. The present study also asked the question of whether language dominance modulates this effect, by exploring whether the size of the trochaic preference is related to the relative amount of German heard, as estimated through parental language reports.

## Experiment

### Methods

#### Ethical Statement

This study was authorized by the ethics committee “Comité de Protection des Personnes Ile de France II” (decision 2011 06).

#### Participants

Twenty-four French/German 6-month-olds (*M* = 6;6; range: 6;00–7;4; 16 girls and 8 boys), were tested in Paris. All participants were born full term, without apparent health problems. They were recruited from birth-lists obtained through the Paris city hall archives and from the “CAFE bilingue,” an association for the promotion of bilingual education. Informed written consent was obtained from all parents. The infants’ relative exposure to their two languages, both within and outside (e.g., extended family, caregivers and friends) the nuclear family, was measured using the Language Exposure Questionnaire (Bosch and Sebastián-Gallés, 1997). Only infants exposed to both French and German between 30 to 70% of the time, and to no other languages, were included in the study. Mean exposure to German was 53.7%. Two additional infants were tested but did not complete the experiment due to fussiness.

#### Stimuli

The stimuli were those used in Höhle et al. (2009). They consisted of CVCV /gaba/ sequences, stressed either on the first syllable (trochaic pattern) or on the second syllable (iambic pattern). Several tokens of each stress pattern were recorded by a German female native speaker. The first syllables of the trochaic sequences had a mean duration of 283 ms (*SD* = 20.8), the second syllables of the trochaic sequences one of 308 ms (*SD* = 25.0). The analysis of pitch revealed an average of 195 Hz (*SD* = 3.9) on the first and 163 Hz (*SD* = 15.9) on the second syllables. The first syllables of the iambic sequences had an average duration of 173 ms (*SD* = 11.0) whereas the second syllables had a mean duration of 430 ms (*SD* = 21.2). The average pitch of the first syllables was 186 Hz (*SD* = 5.2), that of the second syllables 183 Hz (*SD* = 5.9).

Again following Höhle et al. (2009), the tokens were used to create six files for each stress pattern that differed in the order of presentation of the different tokens, the tokens in a file being separated by pauses of about 600 ms. The trochaic speech files contained 16 tokens and had an average duration of 18.39 s (range: 18.28–18.51 s) and the iambic files contained 15 tokens and had an average duration of 18.01 s (range: 18.00–18.07 s). The difference in number of tokens per file is due to the fact that the iambic bisyllables had a slightly longer average duration (603 ms) than the trochaic bisyllables (591 ms).

#### Procedure, Apparatus, and Design

We used the Headturn Preference Paradigm (HPP) as introduced by Hirsh-Pasek et al. (1987). The procedure, apparatus and design were the same as for the monolingual French infants in Experiment 3 of Höhle et al. (2009).

The experiment was run by a Dell Optiplex computer. During the experimental session, the infant was seated on the lap of a caregiver in the center of a test booth. The caregiver listened to music over headphones to prevent influences on the infant’s behavior. Furthermore, he or she was instructed not to interfere with the infant’s behavior during the experiment. Inside the booth, three lamps were fixated: a green one at the center wall, and red ones at each of the side walls. Directly above the green lamp on the center wall was a hole for the lens of a video-camera. On the inside of the test booth, two loudspeakers (SONY xs-F1722) were mounted just below the red side lamps. Each experimental trial started by the blinking of the green center lamp. When the infant oriented to the green lamp, this lamp went out and one of the red lamps on a side wall started to blink. When the infant turned her head toward the red lamp, the speech stimulus was presented from the loudspeaker on the same side as the blinking red lamp. The trial ended when the infant turned her head away for more than 2 s, or when the end of the speech file was reached. If the infant turned away for less than 2 s, the presentation of the speech file continued but the time spent looking away was not included in the total listening time.

The first two speech files – one of the trochaic and one of the iambic pattern – served as warm-up trials and were not included in the data analysis. The remaining 12 experimental speech files were presented in random order. The duration of each experimental session lasted approximately 3–5 min.

## Results

**FIGURE 1:**
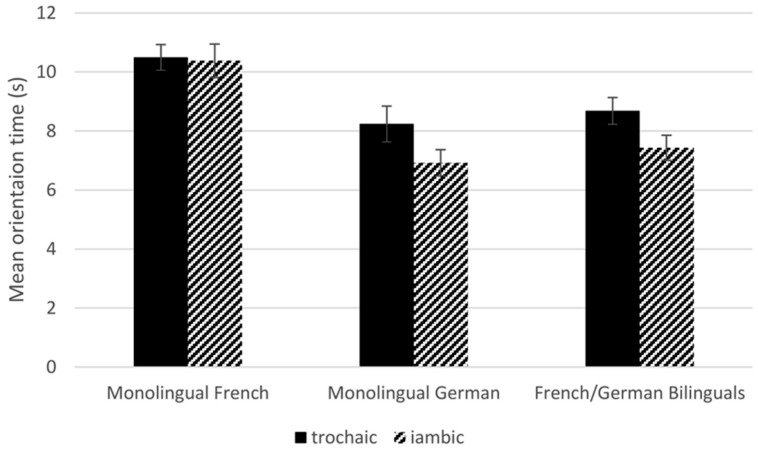
**Mean orientation times (s) to the trochaic and iambic stimuli in 6-month-old infants: monolingual French and German from Höhle et al. (2009) and French/German bilinguals from the present study**.

As for the experiments in Höhle et al. (2009), all individual orientation times exceeding 18 s were reduced to 18 s; two trials were cut off, accounting for 0.7% of all trials. Mean orientation times for each of the two rhythmic patterns were calculated for each infant. On average, infants oriented for 8.68 s (*SE* = 0.45) to the trochaic sequences and for 7.43 s (*SE* = 0.42) to the iambic sequences (see **Figure 1**). This difference was significant, *t*(23) = 2.43, *p* = 0.02, two-tailed, Cohen’s *d* = 1.01, large effect. Sixteen of the 24 infants had longer orientation times to the trochaic than to the iambic sequences. In order to evaluate whether the size of the trochaic bias was influenced by infants’ amount of exposure to German, the difference in orientation times for trochaic minus iambic stimuli was correlated with their percentage of German input. No significant correlation was found, *r* = –0.12, *p* = 0.58.

## Discussion

The aim of the present study was to evaluate the emergence of a trochaic bias in simultaneous French/other language bilinguals, given prior evidence that such a bias emerges in several stress-based languages (German, English), but not in French (Jusczyk et al., 1993; Höhle et al., 2009). Our study used the exact same procedure and German stimuli as Höhle et al. (2009), which had found the emergence of a trochaic bias in monolingual German-learning infants between 4 and 6 months, but no preference in monolingual French-learning infants at 6 months. In this context, the finding of a trochaic preference in French/German bilingual 6-month-olds establishes for the first time that there is no delay in this area of prosodic acquisition for this bilingual population compared to German-learning monolinguals. Moreover, the fact that performance was not affected by the relative amount of exposure to the two languages suggests that even 30% of exposure to German is enough for bilingual infants to develop a trochaic bias that can be used when processing German stimuli.

In our previous studies, we had found that at 10 months of age, bilingual infants learning French and a language with lexical stress do not show the same decline in their sensitivity to stress contrasts than monolingual French-learners, and that they remain sensitive to such contrasts in contexts either lacking (Bijeljac-Babic et al., 2012) or incorporating (Abboub et al., 2015) segmental variability. This sensitivity to stress information constitutes a prerequisite to be able to process lexical stress information in their speech input, and thus be able to discover the predominant lexical stress pattern of the languages spoken in their environment. Taken together with the current findings, this suggests that bilingual infants’ exposure to one language with lexical stress not only maintains sensitivity to this dimension but also provides them with a basis for learning its predominant prosodic pattern without any delay compared to monolinguals.

These findings, however, do not necessarily mean that French-“lexical stress language” bilinguals will process prosodic information as well as “variable stress language” monolinguals later in life. Indeed Dupoux et al. (2010) found that simultaneous French–Spanish bilingual adults had stress processing performance that fell in between that found for Spanish- and French-learning monolinguals. Moreover, specific experience with an L2 with variable stress was found to increase performance in a task in which French speakers learning an L2 with variable lexical stress were judging whether syllable sequences were heard as made up of trochaic or iambic syllable pairs (Boll-Avetisyan et al., 2015) but not when they had to discriminate pairs of syllables with different stress patterns (Dupoux et al., 2008). Whether these differential effects are due to linguistic differences (e.g., in the L2 learned -German versus Spanish-, or the level of processing taped by the experimental task used utterance vs. word) or the way experience with L2 was evaluated (see Boll-Avetisyan et al., 2015, for more detailed discussion) will need to be further explored. Such future studies will help specify the circumstances in which these early prosodic acquisitions in bilingual infants will (or will not) translate into efficient prosodic processing in adulthood.

Relatedly, one intriguing aspect of the present findings is the lack of an effect of the relative language exposure. Indeed, in our study, infants were hearing between 30 and 70% of German according to our estimation using a detailed language questionnaire. This means that only hearing 30% of German was apparently enough for these bilingual infants to acquire a trochaic bias at around the same age at which monolingual infants with a 100% of exposure to German show this bias. How can we reconcile these findings? First, it should be noted that little is known about the impact of language dominance on early language processing and acquisition. To the best of our knowledge, only one study explored the role of dominance for the processing of segmental information, and more precisely the acquisition of the phonotactic properties of the native language (Sebastián-Gallés and Bosch, 2002). They found that both Catalan-learning monolingual and Catalan-dominant Catalan/Spanish bilingual 11-month-olds had learned phonotactic properties of Catalan, while a similar but non-significant effect was found in Spanish-dominant Catalan/Spanish bilinguals of the same age, suggesting a weak dominance effect. For prosody, our two previous studies on stress pattern discrimination by 10-month-old bilinguals also revealed little to no effect of language dominance. Indeed, no impact of language dominance was found when using stimuli with segmental variability (Abboub et al., 2015). Moreover, the only marginal discrimination effect for the more balanced French/German bilinguals tested with materials lacking segmental variability compared to the significant effect of the German dominant bilinguals (Bijeljac-Babic et al., 2012) may reach significance by increasing statistical power when testing a higher number of infants. Overall, the existing evidence so far does not provide strong evidence that language dominance has large effects on infants’ speech processing and their early phonological development.

So how could bilingual infants learn phonological properties of their native language at the same age as their monolingual peers, even though they very likely receive less input in each of their languages? One possibility is that the acquisition of linguistic properties requires not only a certain amount of exposure (and in the present case, 30% or more of German would be enough for bilinguals to get enough input), but also a certain amount of exposure time over development in order to detect and learn properties of the native language. This duration of exposure factor might be related to the need for some flexibility in acquisition (in order not to learn too quickly erroneous properties, or to be able to unlearn an acquired property if it happens to be erroneous). It might also be linked to the need for certain neural networks, linguistic or cognitive abilities, to be set into place or reach a certain maturation level before phonological acquisition can take place. While this hypothesis would need to be tested empirically, note that it might be indirectly supported by data showing that while 6- and 8-month-old infants can learn a new consonant contrast in 2 min in the lab (Maye et al., 2002), it takes them around 8–10 months to learn native consonant categories in the real world (Werker and Tees, 1984; Kuhl et al., 1992), time during which they probably accumulate much more input than in the Maye et al. (2002) experiment. In this perspective, it would mean that the 30–70% of German input that our bilingual infants are receiving in the 6 first months of their lives is enough for them to learn that German words are predominantly trochaic. Note that this possible importance of duration of exposure might be more relevant for the acquisition of phonological properties than the acquisition of the lexicon, a domain of acquisition in which clear effects of quantity of input have been found (Hurtado et al., 2008), although certainly much more work on this issue will be needed, in both monolingual and bilingual populations.

A second reason for why our French/German bilingual infants were able to specify the predominant stress pattern of German within the same timeframe as German-learning monolinguals, related to bilingual acquisition *per se*, could be that bilingual infants have enhanced cognitive abilities, possibly as a result of hearing two languages at the same time. These enhanced abilities would allow them to learn properties of their native language more rapidly and with less input than needed by monolinguals. Evidence for such advantage has been found in early development, at 18 months of age in memory generalization studies (Brito and Barr, 2012), at 7 months in tasks requiring the acquisition of new linguistic rules (Kovács and Mehler, 2009), or at 6 months of age in studies measuring visual habituation as an index of efficiency in stimulus encoding (Singh et al., 2015). However, some authors have recently argued that the strength of this cognitive advantage in bilinguals remains to be confirmed, and its neural/cognitive bases specified (Costa and Sebastian-Galles, 2014). Future studies will then have to continue exploring these early language dominance effects, keeping in mind the difficulty of evaluating language input, and thus possibly using recording tools such as the LENA system (Oller et al., 2010) to quantify language dominance more objectively than by relying on parental reports.

The present findings also raise several questions regarding the specificity and generality of the prosodic acquisition trajectory found in our bilingual population. First, future studies should explore how the link between the prosodic properties of the two languages in acquisition impacts the developmental pattern that we uncovered. In the present case, French/German bilingual infants have to learn that one of their languages does not have lexical stress, while the other one has variable but predominantly trochaic lexical stress. The learning situation might be different, and lead to a different developmental trajectory, for infants learning two languages that both have lexical stress, but in different positions within the words (that is for example, word-initial vs. word-final). This situation could constitute a more difficult acquisition context that might lead to delayed acquisition since infants would have to learn two different stress pattern assignment rules, rather than just one. Indeed, it remains to be determined whether some variation in the developmental trajectory related to early bilingual exposure can be found for the acquisition of prosodic properties, as has been found for the acquisition of segmental properties (e.g., Bosch and Sebastián-Gallés, 2003; Sebastián-Gallés and Bosch, 2009). Second, if the bias observed in the present study results from the acquisition of a language with trochaic lexical stress (German), then it should be observed in French/other language bilinguals if and only if their other language gives rise to a trochaic bias. In order to explore this prediction, we are in the process of testing bilingual infants, all learning French and a language other than German, separating these bilinguals depending on whether their second language would result in the acquisition of a trochaic bias or not.

Third, it will be of interest to determine whether the trochaic bias found in French/German bilingual 6-month-olds in the present experiment only applies to German stimuli, or whether it would also be found if such bilinguals were presented with stimuli that have segmental properties typical for French but not for German. Since infants theoretically can discriminate French and German from birth as these two languages have different rhythms (Mehler et al., 1988; Nazzi et al., 1998), they should be able to learn separate properties of these two languages and use them in language-appropriate ways already at 6 months of age.

## Conclusion

The present study establishes the acquisition of a trochaic bias in French/German bilingual infants at 6 months of age, the same age at which this prosodic development has been found in monolingual infants (Höhle et al., 2009). This first study exploring the acquisition of a prosodic property by bilingual infants thus establishes that this acquisition is not delayed by bilingualism. Following up on this, it will be of interest to further explore the specificity of this developmental trajectory (as discussed above), and also its scope, in particular whether the acquisition of the trochaic bias in these bilingual infants will have implications at higher levels of linguistic processing. More specifically, it will be of interest to explore word form segmentation abilities in bilingual infants given evidence that the emergence of word form segmentation abilities is language-specific in monolingual infants, being based on the stress pattern in trochaic dominant languages (for English and Dutch: Jusczyk et al., 1999; Kooijman et al., 2005, 2009) but on the syllable in French (Nazzi et al., 2006; Goyet et al., 2010, 2013; Nishibayashi et al., 2015).

## Author Contributions

All authors designed the experiment, analyzed and discussed the results and contributed to the writing of the paper. RB-B organized the testing of the infants.

## Conflict of Interest Statement

The authors declare that the research was conducted in the absence of any commercial or financial relationships that could be construed as a potential conflict of interest.

## References

[B1] Abboub N. Bijeljac-Babic R. Serres J. Nazzi T. (2015). On the importance of being bilingual: word stress processing in a context of segmental variability. *J. Exp. Child Psychol.* 132 111–120. 10.1016/j.jecp.2014.12.004 25644083

[B2] Albareda-Castellot B. Pons F. Sebastian-Galles N. (2011). The acquisition of phonetic categories in bilingual infants: new data from an anticipatory eye movement paradigm. *Dev. Sci.* 14 395–401. 10.1111/j.1467-7687.2010.00989.x 22213908

[B3] Bhatara A. Boll-Avetisyan N. Agus T. Höhle B. Nazzi T. (2015). Language experience affects grouping of musical instrument sounds. *Cogn. Sci.* 10.1111/cogs.12300 [Epub ahead of print]. 26480958

[B4] Bhatara A. Boll-Avetisyan N. Unger A. Nazzi T. Höhle B. (2013). Native language and stimulus complexity affect rhythmic grouping of speech. *J. Acoust. Soc. Am.* 134 3828–3843. 10.1121/1.4823848 24180792

[B5] Bijeljac-Babic R. Serres J. Höhle B. Nazzi T. (2012). Effect of bilingualism on lexical stress pattern discrimination in French-learning infants. *PLoS ONE* 7:e30843 10.1371/journal.pone.0030843 PMC3281880 22363500

[B6] Boll-Avetisyan N. Bhatara A. Unger A. Nazzi T. Höhle B. (2015). Effects of experience with L2 and music on rhythmic grouping by French listeners. *Biling. Lang. Cogn.* (in press). 10.1017/S1366728915000425

[B7] Bosch L. Sebastián-Gallés N. (1997). Native-language recognition abilities in four month-old infants from monolingual and bilingual environments. *Cognition* 65 33–69. 10.1016/S0010-0277(97)00040-1 9455170

[B8] Bosch L. Sebastián-Gallés N. (2003). Simultaneous bilingualism and the perception of a language-specific vowel contrast in the first year of life. *Lang. Speech* 46 217–243. 10.1177/00238309030460020801 14748445

[B9] Brito N. Barr R. (2012). Influence of bilingualism on memory generalization during infancy. *Dev. Sci.* 15 812–816. 10.1111/j.1467-7687.2012.1184.x 23106735

[B10] Burns T. C. Yoshida K. A. Hill K. Werker J. F. (2007). The development of phonetic representation in bilingual and monolingual infants. *Appl. Psycholinguist.* 28 455–474. 10.1017/S0142716407070257

[B11] Butler J. Floccia C. Goslin J. Panneton R. (2011). Infants’ discrimination of familiar and unfamiliar accents in speech. *Infancy* 16 392–417. 10.1111/j.1532-7078.2010.00050.x 32693512

[B12] Byers-Heinlein K. Burns T. C. Werker J. F. (2010). The roots of bilingualism in newborns. *Psychol. Sci.* 21 343–348. 10.1177/0956797609360758 20424066

[B13] Christophe A. Morton J. (1998). Is Dutch native English? Linguistic analysis by 2-month-olds. *Dev. Sci.* 1 215–219. 10.1111/1467-7687.00033

[B14] Costa A. Sebastian-Galles N. (2014). How does the bilingual experience sculpt the brain? *Nat. Rev. Neurosci.* 15 336–345. 10.1038/nrn3709 24739788 PMC4295724

[B15] Di Cristo A. (1999). Vers une modélisation de l’accentuation du français: première partie. *J. French Lang. Stud.* 9 143–179. 10.1017/S0959269500004671

[B16] Dupoux E. Pallier C. Sebastian-Galles N. Mehler J. (1997). A distressing “deafness” in French? *J. Mem. Lang.* 36 406–421. 10.1006/jmla.1996.2500

[B17] Dupoux E. Peperkamp S. Sebastian-Galles N. (2001). A robust method to study stress “deafness”. *J. Acoust. Soc. Am.* 110 1606–1618. 10.1121/1.1380437 11572370

[B18] Dupoux E. Peperkamp S. Sebastian-Gallés N. (2010). Limits on bilingualism revisited: stress ’deafness’ in simultaneous French-Spanish bilinguals. *Cognition* 114 266–275. 10.1016/j.cognition.2009.10.001 19896647

[B19] Dupoux E. Sebastian-Gallés N. Navarrete E. Peperkamp S. (2008). Persistent stress “deafness”: the case of French learners of Spanish. *Cognition* 106 682–706. 10.1016/j.cognition.2007.04.001 17592731

[B20] Féry C. Hörnig R. Pahaut S. (2011). “Correlates of phrasing in French and German from an experiment with semi-spontaneous speech,” in *Intonational Phrasing in Romance and Germanic: Cross-Linguistic and Bilingual Studies* Vol. 10 eds Gabriel C. Lleo C. (Amsterdam: John Benjamins Publishing), 11–42. 10.1075/hsm.10.03fer

[B21] Friederici A. D. Friedrich M. Christophe A. (2007). Brain responses in 4-month-old infants are already language specific. *Curr. Biol.* 17 1208–1211. 10.1016/j.cub.2007.06.011 17583508

[B22] Garcia-Sierra A. Rivera-Gaxiola M. Percaccio C. R. Conboy B. T. Romo H. Klarman L. (2011). Bilingual language learning: an ERP study relating early brain responses to speech, language input, and later word production. *J. Phonetics* 39 546–557. 10.1016/j.wocn.2011.07.002

[B23] Goyet L. de Schonen S. Nazzi T. (2010). Syllables in word segmentation by French-learning infants: an ERP study. *Brain Res.* 1332 75–89. 10.1016/j.brainres.2010.03.047 20331982

[B24] Goyet L. Nishibayashi L.-L. Nazzi T. (2013). Early syllabic segmentation of fluent speech by infants acquiring French. *PLoS ONE* 8:e79646 10.1371/journal.pone.0079646 PMC3820683 24244536

[B25] Grosjean F. (2010). *Bilingual: Life and Reality*. Cambridge, MA: Harvard University Press.

[B26] Hayes B. (1995). *Metrical Stress Theory: Principles and Case Studies*. Chicago: University of Chicago Press.

[B27] Herold B. Höhle B. Walch E. Weber T. Obladen M. (2008). Impaired stress pattern discrimination in very low birth weight infants during the first six months of life. *Dev. Med. Child Neurol.* 50 678–683. 10.1111/j.1469-8749.2008.03055.x 18754917

[B28] Hirsh-Pasek K. Kemler Nelson D. G. Jusczyk P. W. Wright Cassidy K. Druss B. Kennedy L. (1987). Clauses are perceptual units for young Infants. *Cognition* 26 269–286. 10.1016/S0010-0277(87)80002-1 3677573

[B29] Höhle B. Bijeljac-Babic R. Herold B. Weissenborn J. Nazzi T. (2009). The development of language specific prosodic preferences during the first half year of life: evidence from German and French. *Infant Behav. Dev.* 2 262–274. 10.1016/j.infbeh.2009.03.004 19427039

[B30] Höhle B. Pauen S. Hesse V. Weissenborn J. (2014). Discrimination of rhythmic pattern at 4 months and language performance at 5 years: a longitudinal analysis of data from German-learning infants. *Lang. Learn.* 64 141–164. 10.1111/lang.12075

[B31] Hurtado N. Marchman V. A. Fernald A. (2008). Does input influence uptake? Links between maternal talk, processing speed and vocabulary size in Spanish-learning children. *Dev. Sci.* 11 F31–F39. 10.1111/j.1467-7687.2008.00768.x 19046145 PMC2898277

[B32] Jun S. A. Fougeron C. (2000). “A phonological model of French intonation,” in *Intonation: Analysis, Modeling and Technology*, ed. Botinis A. (Dordrecht: Kluwer Academic Press), 209–242.

[B33] Jun S. A. Fougeron C. (2002). Realizations of accentual phrase in French intonation. *Probus* 14 147–172. 10.1515/prbs.2002.002

[B34] Jusczyk P. W. Cutler A. Redanz N. J. (1993). Preference for the predominant stress patterns of English words. *Child Dev.* 64 675–687. 10.2307/1131210 8339688

[B35] Jusczyk P. W. Houston D. M. Newsome M. (1999). The beginning of word segmentation in English-learning infants. *Cogn. Psychol.* 39 159–207. 10.1006/cogp.1999.0716 10631011

[B36] Kooijman V. Hagoort P. Cutler A. (2005). Electrophysiological evidence for prelinguistic infants’ word recognition in continuous speech. *Cogn. Brain Res.* 24 109–116. 10.1016/j.cogbrainres.2004.12.009 15922163

[B37] Kooijman V. Hagoort P. Cutler A. (2009). Prosodic structure in early word segmentation: ERP evidence from Dutch ten-month-olds. *Infancy* 6 591–612. 10.1080/15250000903263957 32693518

[B38] Kovács A. Mehler J. (2009). Cognitive gains in 7-month-old bilingual infants. *Proc. Natl. Acad. Sci. U.S.A.* 106 6556–6560. 10.1073/pnas.0811323106 19365071 PMC2672482

[B39] Kuhl P. K. Williams K. A. Lacerda F. Stevens K. N. Lindblom B. (1992). Linguistic experience alters phonetic perception in infants by 6 months of age. *Science* 255 606–608. 10.1126/science.1736364 1736364

[B40] Maye J. Werker J. F. Gerken L. (2002). Infant sensitivity to distributional information can affect phonetic discrimination. *Cognition* 82 B101–B111. 10.1016/S0010-0277(01)00157-3 11747867

[B41] Mehler J. Jusczyk P. W. Lambertz G. Halsted N. Bertoncini J. Amiel-Tison C. (1988). A precursor of language acquisition in young infants. *Cognition* 29 143–178. 10.1016/0010-0277(88)90035-2 3168420

[B42] Michelas A. D’Imperio M. (2015). Prosodic boundary strength guides syntactic parsing of French utterances. *Lab. Phonol.* 6 119–146. 10.1515/lp-2015-0003

[B43] Molnar M. Gervain J. Carreiras M. (2013). Within-rhythm class native language discrimination abilities of Basque-Spanish monolingual and bilingual infants at 3.5 months of age. *Infancy* 19 326–337. 10.1111/infa.12041

[B44] Moon C. Panneton-Cooper R. Fifer W. P. (1993). Two-day-olds prefer their native language. *Infant Behav. Dev.* 16 495–500. 10.1016/0163-6383(93)80007-U

[B45] Nazzi T. Bertoncini J. Mehler J. (1998). Language discrimination by newborns: towards an understanding of the role of rhythm. *J. Exp. Psychol.* 24 756–776. 10.1037//0096-1523.24.3.756 9627414

[B46] Nazzi T. Iakimova G. Bertoncini J. Frédonie S. Alcantara C. (2006). Early segmentation of fluent speech by infants acquiring French: emerging evidence for crosslinguistic differences. *J. Mem. Lang.* 54 283–299. 10.1371/journal.pone.0079646

[B47] Nazzi T. Jusczyk P. W. Johnson E. K. (2000). Language discrimination by English learning 5-month-olds: effects of rhythm and familiarity. *J. Mem. Lang.* 43 1–19. 10.1006/jmla.2000.2698

[B48] Nespor M. Shukla M. van de Vijver R. Avesani C. Schraudolf H. Donati C. (2008). Different phrasal prominence realization in VO and OV languages. *Lingue Linguaggio* 7 1–28.

[B49] Nishibayashi L.-L. Goyet L. Nazzi T. (2015). Early speech segmentation in French-learning infants: monosyllabic words versus embedded syllables. *Lang. Speech* 58 334–350. 10.1177/0023830914551375 26529900

[B50] Oller D. K. Niyogi P. Gray S. Richards J. A. Gilkerson J. Xu D. (2010). Automated vocal analysis of naturalistic recordings from children with autism, language delay, and typical development. *Proc. Natl. Acad. Sci. U.S.A.* 27 13354–13359. 10.1073/pnas.1003882107 20643944 PMC2922144

[B51] Pons F. Bosch L. (2010). Stress pattern preference in Spanish-learning infants: the role of syllable weight. *Infancy* 15 223–245. 10.1111/j.1532-7078.2009.00016.x 32693542

[B52] Sansavini A. Bertoncini J. Giovanelli G. (1994). Newborns discriminate stress patterns in phonetically complex Italian words. *Infant Behav. Dev.* 17:924.

[B53] Sansavini A. Bertoncini J. Giovanelli G. (1997). Newborns discriminate the rhythm of multisyllabic stressed words. *Dev. Psychol.* 33 3–11. 10.1037/0012-1649.33.1.3 9050385

[B54] Sebastián-Gallés N. Bosch L. (2002). Building phonotactic knowledge in bilinguals: role of early exposure. *J. Exp. Psychol.* 28 974–989. 12190262

[B55] Sebastián-Gallés N. Bosch L. (2009). Developmental shift in the discrimination of vowel contrasts in bilingual infants: is the distributional account all there is to it? *Dev. Sci.* 12 874–887. 10.1111/j.1467-7687.2009.00829.x 19840043

[B56] Singh L. Fu C. S. L. Rahman A. A. Hameed W. B. Sanmugam S. Agarwal P. (2015). Back to basics: a bilingual advantage in infant visual habituation. *Child Dev.* 86 294–302. 10.1111/cdev.12271 25074016

[B57] Skoruppa K. Cristià A. Peperkamp S. Seidl A. (2011). English-learning infants’ perception of word stress patterns. *J. Acoust. Soc. Am.* 130 EL50–EL55. 10.1121/1.3590169 21786868

[B58] Skoruppa K. Pons F. Bosch L. Christophe A. Cabrol D. Peperkamp S. (2013). The development of word stress processing in French and Spanish infants. *Lang. Learn. Dev.* 9 88–104. 10.1080/15475441.2012.693881

[B59] Skoruppa K. Pons F. Christophe A. Bosch L. Dupoux E. Sebastian-Galles N. (2009). Language-specific stress perception by nine-month-old French and Spanish infants. *Dev. Sci.* 12 914–919. 10.1111/j.1467-7687.2009.00835.x 19840046

[B60] Spring D. R. Dale P. S. (1977). Discrimination of linguistic stress in early infancy. *J. Speech Hear. Res.* 20 224–232. 10.1044/jshr.2002.224 895094

[B61] Sundara M. Polka L. Molnar M. (2008). Development of coronal stop perception: bilingual infants keep pace with their monolingual peers. *Cognition* 108 232–242. 10.1016/j.cognition.2007.12.013 18281027

[B62] Sundara M. Scutellaro A. (2011). Rhythmic distance between languages affects the development of speech perception in bilingual infants. *J. Phonet.* 39 505–513. 10.1016/j.wocn.2010.08.006

[B63] Vaissière J. Michaud A. (2006). “Prosodic constituents in French: a data-driven approach,” in *Prosody and Syntax*, eds Fonagy I. Kawaguchi Y. Moriguchi T. (Amsterdam: John Benjamins Publishing), 47–64.

[B64] Werker J. F. Tees R. C. (1984). Cross-language speech perception: evidence for perceptual reorganization during the first year of life. *Infant Behav. Dev.* 7 49–63. 10.1016/S0163-6383(84)80022-3

